# Severe community-acquired adenovirus pneumonia in an immunocompetent 44-year-old woman: a case report and review of the literature

**DOI:** 10.1186/1752-1947-5-259

**Published:** 2011-06-30

**Authors:** Tristan W Clark, Daniel H Fleet, Martin J Wiselka

**Affiliations:** 1 Department of Infectious Diseases, Leicester Royal Infirmary, Level 6 Windsor Building, Leicester, LE1 5WW, UK

## Abstract

**Introduction:**

This case report describes a rare condition: community-acquired adenovirus pneumonia in an immunocompetent adult. The diagnosis was achieved by using a multiplex real-time reverse transcriptase polymerase chain reaction (RT-PCR) assay and highlights the usefulness of these novel molecular diagnostic techniques in patients hospitalized with acute respiratory illness. We also performed a literature search for previously published cases and present a summary of the clinical, laboratory and radiological features of this condition.

**Case presentation:**

A 44-year-old immunocompetent Caucasian woman was admitted to our hospital with an acute febrile respiratory illness associated with a rash. Her blood tests were non-specifically abnormal, and tests for bacterial pathogens were negative. Her condition rapidly deteriorated while she was in our hospital and required mechanical ventilation and inotropic support. A multiplex real-time RT-PCR assay performed on respiratory specimens to detect respiratory viruses was negative for influenza but positive for adenovirus DNA. The patient recovered on supportive treatment, and antibiotics were stopped after 5 days.

**Conclusions:**

Community-acquired adenovirus pneumonia in immunocompetent adult civilians presents as a non-specific acute febrile respiratory illness followed by the abrupt onset of respiratory failure, often requiring mechanical ventilation. Its laboratory and radiological features are typical of viral infections but also are non-specific. Novel multiplex real-time RT-PCR testing for respiratory viruses enabled us to rapidly make the diagnosis in this case. The new technology could be used more widely in patients with acute respiratory illness and has potential utility for rationalization of the use of antibiotics and improving infection control measures.

## Introduction

Adenoviruses are double-stranded DNA viruses belonging to the family *Adenoviridae*. There are over 50 known serotypes of adenovirus, which are categorized into six subgenera (A to F). Adenoviruses are a common cause of acute febrile and respiratory infections in children and are generally self-limiting [1]. Severe infections, including pneumonia, can occur in neonates [2] and in adults with compromised immunity, such as those with hematopoietic stem cell transplants and in patients with human immunodeficiency virus (HIV) infection [3]. Outbreaks of acute respiratory illness, including pneumonia, caused by adenovirus serotypes 3, 4, 7, 14 and 21 are common among military recruits, and fatal outcomes have occasionally been reported [4-6]. Outbreaks of adenovirus infection in long-term nursing facilities and in hospital wards with associated cases of fatal pneumonia have also been described [7]. In contrast, community-acquired adenovirus pneumonia in immunocompetent adult civilians has rarely been described. We report the case of a previously healthy and immunocompetent woman with severe adenovirus pneumonia who developed rapidly progressive respiratory failure requiring mechanical ventilation and who made a successful recovery after being treated with supportive measures. We also summarize the demographic, clinical, laboratory and radiological features of community-acquired adenovirus pneumonia cases in immunocompetent adult civilians that have previously been reported in the literature.

## Case presentation

A 44-year-old Caucasian woman was admitted to our emergency department with a three-day history of a febrile illness associated with sore throat, dry cough, myalgia and diarrhea. One day prior to admission she had developed a widespread, non-pruritic, erythematous rash. Her medical history consisted of hypertension, for which she was taking atenolol, and several episodes of gout, for which she was taking allopurinol.

Her physical examination revealed that she was obese, had a body temperature of 39.0°C, a pulse rate of 112beats/minute and blood pressure of 145/90 mmHg. Her respiratory rate was 20 breaths/minute with oxygen saturation of 94% on room air. Her chest auscultation was unremarkable. She had a widespread, erythematous maculopapular rash with scattered petechiae on both legs. Examination of the oropharynx revealed erythema but no exudate.

Initial laboratory tests showed a white cell count of 9.2 × 10^9^/L, a neutrophil count of 7.9 × 10^9^/L, a lymphocyte count of 0.69 × 10^9^/L, a platelet count of 254 × 10^9^/L, a C-reactive protein concentration of 169 mg/L, an alanine aminotransferase level of 22 IU/mL, a creatinine phosphokinase (CPK) level of 950 IU/mL and a creatinine concentration of 73 μmol/L. Her HIV test was negative. Her anti-nuclear antibodies, rheumatoid factor and anti-neutrophil cytoplasmic antibodies were negative, and her complement components C3 and C4 and immunoglobulin levels were within the normal range. Her initial chest radiograph was unremarkable. She was commenced on intravenous ceftriaxone for presumed meningococcal disease.

**Figure 1:**
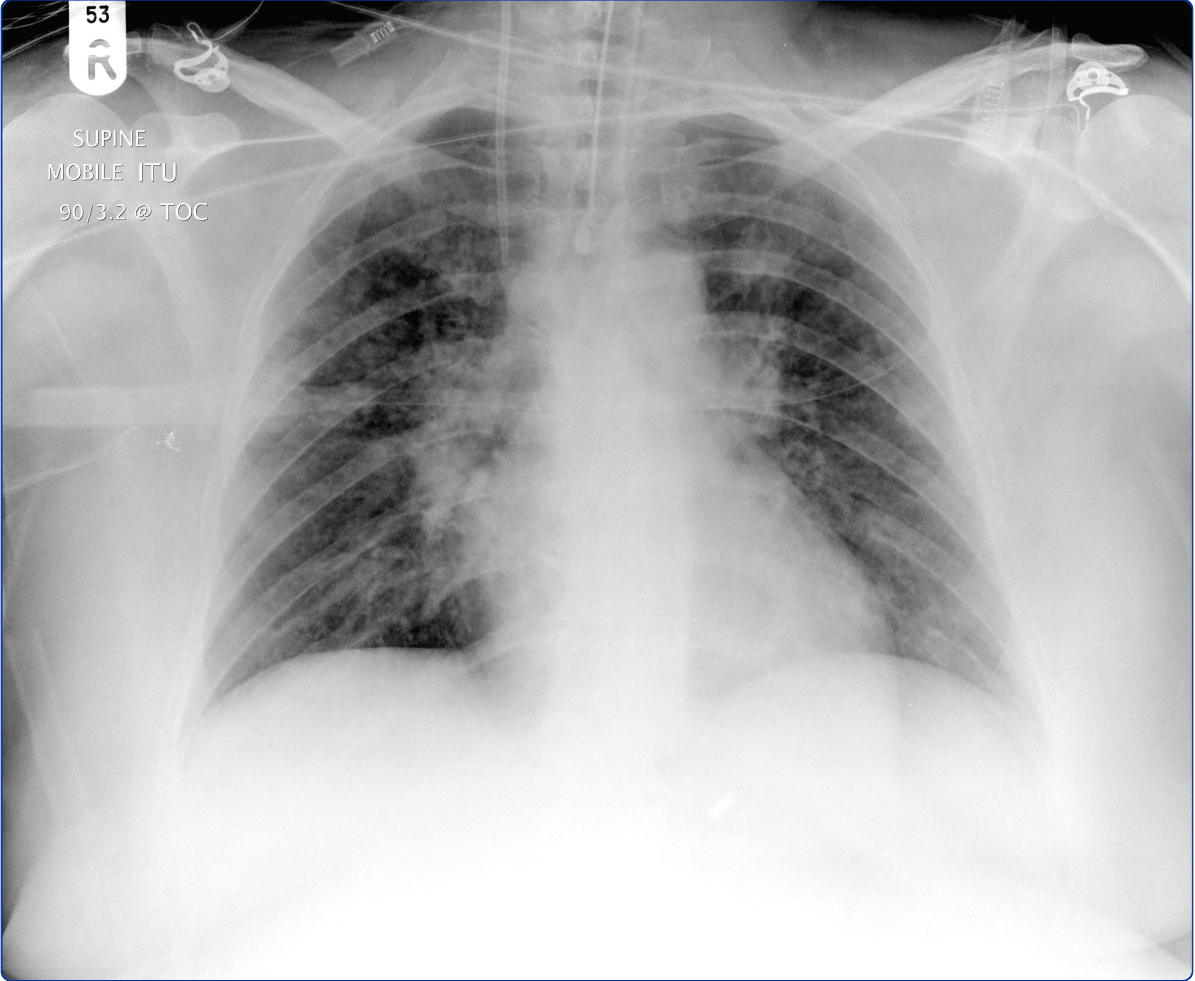
**The patient's repeat chest radiograph showing widespread bilateral interstitial infiltrates**.

Twenty-four hours following admission her condition rapidly deteriorated with acute respiratory failure and hypotension requiring admission to the intensive care unit for mechanical ventilation and vasopressor support. A repeat chest radiograph showed widespread interstitial infiltrates bilaterally (Figure 1). Her antibiotics were changed to imipenem and doxycycline to treat presumed bacterial pneumonia, and oseltamivir was empirically added to treat a possible 2009 pandemic influenza A (H1N1) infection.

Bacterial cultures of her blood and sputum, *Legionella *antigen testing of her urine, and a polymerase chain reaction (PCR) assay of her blood for *Neisseria meningitidis *and *Streptococcus pneumoniae *were all negative. Her nasopharyngeal and tracheal samples were negative for influenza A and B (including H1N1), respiratory syncytial virus (RSV) types A and B and parainfluenza virus (PIV) types 1 through 4, but they were positive for adenovirus DNA on the basis of PCR assay (using the hexon gene as the target for amplification), with a cycle threshold value of 18. Subsequent sequencing analysis performed at the respiratory Virus Reference Laboratory, London, revealed the isolate to belong to serotype 4.

The patient made an uncomplicated recovery without any specific antiviral therapy and was extubated on the fifth day of her admission. Antibiotics were stopped after a total of five days, and she was discharged to home on the ninth day of her admission. Further tests for immunodeficiency were negative.

We performed a literature search of MEDLINE for cases of community-acquired adenovirus pneumonia in immunocompetent adults. We used the search terms "adenovirus," "pneumonia," "immunocompetent," "adult" and "civilian." We excluded cases that involved military recruits, nosocomial cases and those cases in which bacterial pathogens were also implicated.

We identified 19 articles published between 1975 and 2008 [8-26] describing 21 patients that matched our search terms. The demographic, laboratory, radiological and clinical details of these cases and our own are shown in Table 1.

**Table 1:** The demographic, clinical, laboratory, radiological and outcome data for the 21 cases reported in the literature and in our patient^a^

Patient characteristics	Our case	Previously reported cases (*n *= 21)
Demographics		
Age, yr	44	40 [18 to 60]
Sex	Female	Male 12 (57)
Presenting symptoms		
Preceding history, days	3	5 [1 to 28]
Fever	Yes	19 (90)
Cough	Yes	17 (81)
Dyspnea	Yes	15 (70)
Myalgia	Yes	11 (57)
Sore throat	Yes	6 (29)
Abdominal pain	Yes	3 (14)
Diarrhea	Yes	2 (10)
Examination findings		
Fever (temperature > 38°C)	Yes	17 (81)
Chest signs	No	19 (90)
Hypoxia	No	16 (66)
Pharyngitis	Yes	4 (19)
Conjunctivitis	No	4 (19)
Rash	Yes	1 (5)
Laboratory tests		
Total white cell count (4 to 11 × 10^9^/L)	6.6	7.7 [3.9 to 28]
Neutrophilia (> 7 × 10^9^/L)	No	7 (33)
Lymphopenia (< 1.0 × 10^9^/L)	Yes	11 (52)
Thrombocytopenia (< 150 × 10^9^/L)	No	4 (19)
Elevated transaminases	No	6 (29)
Elevated CPK	Yes	6 (29)
CXR appearance		
Normal	No	2 (10)
Lobar consolidation	No	5 (24)
Bilateral interstitial infiltrates	Yes	12 (57)
Clinical course/outcome		
Required ET intubation	Yes	14 (67)
Time to Intubation, days	3	1.5 [0.25 to 8]
Treatment with cidofovir	No	2 (10)
Length of hospital stay, days	9	21 [3 to 123]
Died	No	5 (24)
Adenovirus serotype		
3	No	4 (19)
4	Yes	2 (10)
7	No	5 (24)
21	No	3 (14)
Unknown	No	7 (33)

^a^CPK, creatinine phosphokinase; CXR, Chest X ray; ET, endotracheal tube.

Data are expressed as *n *(%) or median [range].

Of the 21 cases retrieved in our literature search, 57% of the patients were men, and overall the patients' median age was 40 years (age range, 18 to 60 years). Where recorded, the commonest ethnic origin of patients was Caucasian (40%). Significant co-morbidity was uncommon among patients, but obesity was frequently noted as an examination finding.

The median duration of illness prior to admission to the hospital was five days. The following presenting symptoms were noted: fever (90%), cough (81%), dyspnea (70%), myalgia (57%), sore throat (29%), abdominal pain (14%) and diarrhea (10%). Common examination findings on presentation included abnormalities in chest auscultation (90%), pyrexia (89%) and hypoxia (66%). The presence of pharyngitis, conjunctivitis or rash was noted infrequently (19%, 19% and 5% respectively).

The median white cell count on admission to the hospital was 7.7 × 10^9 ^(range, 3.9 × 10^9 ^to 28 × 10^9^), although neutrophilia was relatively common (33%). Lymphopenia and thrombocytopenia were noted in 52% and 19% of patients, respectively. Other frequently noted laboratory abnormalities were mildly elevated transaminases and elevated levels of CPK.

The chest radiograph at presentation was abnormal in 90% of patients. The most common pattern of abnormality was bilateral interstitial infiltrates (57%), although lobar consolidation was also noted reasonably frequently (24%).

Intubation and mechanical ventilation were required in 67% of patients and occurred at a median of one and half days following admission. Overall 24% of patients died. The median length of stay in the hospital was 21 days. Two patients received antiviral therapy with cidofovir, one of whom died.

Where recorded, the most common adenovirus serotypes identified were serotype 7 (24%), serotype 3 (19%), serotype 21 (14%) and serotype 4 (10%). The diagnosis was made most frequently on the basis of lower respiratory tract samples (principally bronchoscopic alveolar lavage fluid and lung biopsy tissue), and viral culture was the most common method of adenovirus detection (76%). There were no cases identified in the literature where molecular methods were used to diagnose adenovirus pneumonia.

## Discussion

Our case report and review of the literature provides the first comprehensive review of community-acquired adenovirus pneumonia in immunocompetent adult civilians. Hakim and Tleyjeh [8] published a case report and literature review of adenovirus pneumonia in immunocompetent adults in 2008; however, their cohort was a mix of civilians, military recruits and healthcare-associated cases.

The 21 cases we identified in the literature demonstrate that patients with adenovirus pneumonia usually present with several days' history of a non-specific febrile respiratory illness. These patients frequently have respiratory compromise with hypoxia at the time of presentation, while the classical features of adenoviral infection, such as pharyngitis, conjunctivitis, rash or diarrhea, are usually absent. The clinical condition of most patients deteriorates rapidly during admission and requires intubation and ventilation, a pattern commonly seen with primary influenza pneumonia [27]. Laboratory findings are also typical of viral infection, with a normal total white cell count, relative lymphopenia, thrombocytopenia and elevated transaminases and CPK being frequently observed. The most commonly seen radiological pattern on admission is widespread bilateral interstitial shadowing, which is consistent with the results reported in a case series describing the radiological appearance of adult patients with confirmed adenoviral pneumonia [28]. It is noteworthy that several patients, including our own case, had normal initial chest radiography results. Lobar consolidation, a pattern considered more suggestive of bacterial infection, was observed in around one-fourth of patients with adenoviral pneumonia. All of these radiological patterns (including normal initial chest radiographs) have been described in patients with primary influenza pneumonia [29-31]. Although the overall mortality rate in this series [8-26] was 24%, only two patients who were reported on after 1979 have died, possibly representing improvement in supportive care over this time period.

Our present case report of an immunocompetent adult civilian patient with sporadic adenoviral pneumonia is the first case to be reported in the literature in which molecular diagnostic methods were used. Nucleic acid detection has the advantages of increased sensitivity and rapid availability of results compared to the conventional diagnostic techniques of viral culture and antigen detection [32]. In addition, multiplex real-time reverse transcriptase PCR (RT-PCR) assays are increasingly being used by diagnostic laboratories to detect a wide range of respiratory viruses in a single reaction. While it is well-recognized that influenza virus and adenovirus can cause pneumonia, there is increasing evidence that other respiratory viruses, such as RSV, human metapneumovirus, PIV, human rhinovirus and human coronavirus play an important role in the etiology of community-acquired pneumonia in adults [33]. The increasingly widespread use of multiplex real-time RT-PCR for the detection of respiratory viruses in clinical practice will allow us to accurately determine the burden of respiratory viral infection in patients with community-acquired pneumonia and may demonstrate that adenoviral pneumonia in immunocompetent adults is more common than previously thought.

The advantages of rapidly diagnosing respiratory viral infection in patients with community-acquired pneumonia include the institution of appropriate infection control measures, the rational use of antibiotics in the absence of bacterial co-pathogens and, in some instances, the use of specific antiviral therapy. Two patients in our series [8-26] received the antiviral agent cidofovir, and while there are no randomized, controlled trials demonstrating its efficacy in adenoviral infection, it has been used successfully in immunocompromised patients with severe adenoviral pneumonia [34].

## Conclusions

Our literature review suggests that community-acquired adenoviral pneumonia in immunocompetent adult civilians presents as a non-specific febrile respiratory illness that progresses rapidly to respiratory failure and often requires mechanical ventilation. The laboratory and radiological findings are typical of viral infection but are also non-specific. Novel respiratory virus real-time RT-PCR testing enabled us to rapidly detect adenovirus as the cause of severe community-acquired pneumonia in our patient.

## Abbreviations

ALT: alanine aminotransferase; ANCA: anti-neutrophil cytoplasmic antibodies; BAL: bronchoscopic alveolar lavage; CPK: creatinine phosphokinase; *C*_t_: cycle threshold; RSV: respiratory syncytial virus; RT-PCR: reverse transcriptase polymerase chain reaction.

## Consent

Written informed consent was obtained from the patient for publication of this case report and any accompanying images. A copy of the written consent is available for review by the Editor-in-Chief of this journal.

## Competing interests

The authors declare that they have no competing interests.

## Authors' contributions

TWC was involved in the design of the study, assisted in the literature search and wrote and revised the manuscript. DF was involved in the design of this study, performed the literature search and assisted in the writing of the manuscript. MJW oversaw the study and assisted with the writing of the manuscript. All authors read and approved the final version of the manuscript.
